# Woody encroachment reduces nutrient limitation and promotes soil carbon sequestration

**DOI:** 10.1002/ece3.1024

**Published:** 2014-03-24

**Authors:** Wilma J Blaser, Griffin K Shanungu, Peter J Edwards, Harry Olde Venterink

**Affiliations:** 1 Institute of Integrative Biology, ETH ZurichUniversitaetsstrasse 16, 8092, Zurich, Switzerland; 2 Zambia Wildlife Authority (ZAWA)Private Bag 1, Chilanga, Zambia

**Keywords:** Chronosequence, land cover change, N:P stoichiometry, plant–soil feedback, savanna, tree–grass interactions

## Abstract

During the past century, the biomass of woody species has increased in many grassland and savanna ecosystems. As many of these species fix nitrogen symbiotically, they may alter not only soil nitrogen (N) conditions but also those of phosphorus (P). We studied the N-fixing shrub *Dichrostachys cinerea* in a mesic savanna in Zambia, quantifying its effects upon pools of soil N, P, and carbon (C), and availabilities of N and P. We also evaluated whether these effects induced feedbacks upon the growth of understory vegetation and encroaching shrubs. *Dichrostachys cinerea* shrubs increased total N and P pools, as well as resin-adsorbed N and soil extractable P in the top 10-cm soil. Shrubs and understory grasses differed in their foliar N and P concentrations along gradients of increasing encroachment, suggesting that they obtained these nutrients in different ways. Thus, grasses probably obtained them mainly from the surface upper soil layers, whereas the shrubs may acquire N through symbiotic fixation and probably obtain some of their P from deeper soil layers. The storage of soil C increased significantly under *D. cinerea* and was apparently not limited by shortages of either N or P. We conclude that the shrub *D. cinerea* does not create a negative feedback loop by inducing P-limiting conditions, probably because it can obtain P from deeper soil layers. Furthermore, C sequestration is not limited by a shortage of N, so that mesic savanna encroached by this species could represent a C sink for several decades.

We studied the effects of woody encroachment on soil N, P, and C pools, and availabilities of N and P to *Dichrostachys cinerea* shrubs and to the understory vegetation. Both N and P pools in the soil increased along gradients of shrub age and cover, suggesting that N fixation by *D. cinerea* did not reduce the P supply. This in turn suggests that continued growth and carbon sequestration in this mesic savanna ecosystems are unlikely to be constrained by nutrient limitation and could represent a C sink for several decades.

## Introduction

During the past century, many savannas and grass-dominated ecosystems around the world have been affected by the spread of woody plants (Archer et al. 1995; Van Auken 2000, 2009). Many possible drivers have been proposed for this phenomenon, including changes in climate, atmospheric CO_2_, herbivory, and fire regime, although the relative importance of different factors probably varies among ecoregions (Archer et al. 1995; Van Auken 2009; Buitenwerf et al. 2012).

Many of the encroaching woody species fix nitrogen (N) symbiotically (Knapp et al. 2008; Boutton and Liao 2010; Eldridge et al. 2011) and therefore have the potential to bring additional N into the ecosystem (Ludwig et al. 2004; Boutton and Liao 2010; Cech et al. 2010). However, as these species usually have a high phosphorus (P) requirement (Binkley et al. 2000; Vitousek et al. 2002), they may increase the availability of soil N relative to P (Hibbard et al. 2001; Cech et al. 2008; Boutton and Liao 2010), which in due course could produce P-limiting conditions. This, in turn, could prevent further N fixation (Crews 1993; Pearson and Vitousek 2001; Binkley et al. 2003; Isaac et al. 2011) and restrict the growth of understory vegetation (Riginos et al. 2009; Van Auken 2009; Sitters et al. 2013). However, such a negative feedback caused by N fixation is not inevitable, and some field studies have found that N-fixing woody species – especially those with a high canopy – actually increase P pools in the top soil (e.g., Geesing et al. 2000; Ludwig et al. 2004; Blaser et al. 2013; Sitters et al. 2013). The source of this additional P is unknown; it could potentially come from deeper soil layers, either taken up by deep roots or transported to the upper soil by hydraulic lift (Scholes and Archer 1997; Ludwig et al. 2004; McCulley et al. 2004; Sitters et al. 2013), or it could be taken up from the surface soil through widely spreading lateral roots (Belsky et al. 1989; Scholes and Archer 1997). In addition, many leguminous plants have a high root phosphatase activity, which may give them an advantage over other plants in acquiring soil P present in an organic form (Houlton et al. 2008; Olde Venterink 2011).

While great efforts have gone into understanding consequences of woody encroachment in North American grassland ecosystems, much less is known about African savanna grasslands (Table 1). However, simulations for Africa semi-arid and mesic savannas predict that woody C_3_ plants will have an increasing competitive advantage over C_4_ grasses as atmospheric CO_2_ concentrations increase (Bond et al. 2003). The widespread replacement of grasses by woody plants could have potentially important effects upon both the structure of the vegetation and its above-ground net primary production (ANPP; Knapp et al. 2008; Barger et al. 2011). Apart from light and water availability, ANPP depends also on the availability of the growth-limiting nutrients N and P (Schimel et al. 1996; Cech et al. 2008). If the growth of N-fixing shrubs is affected by a negative feedback due to declining P availability, then their dominance might be of short duration; however, if encroaching N-fixing shrubs do not induce a negative feedback, they might remain abundant, which could have profound consequences for savanna ecosystems. One potentially beneficial effect could be that the tree-dominated ecosystems continue to sequester C and act as a buffer for increased atmospheric CO_2_ levels. Indeed, as grasslands and savanna ecosystems account for 30–35% of the global terrestrial net primary production (Field et al. 1998), an increase in C input through shrub encroachment and subsequent changes in C storage could have global implications for the earth–atmosphere system (Knapp et al. 2008).

**Table 1:** A survey of studies reporting C, N, and P accretion rates in the topsoil after woody encroachment.

Region	Mean annual precipitation^1^ (mm·year^−1^)	Encroaching species	Stand age (years)	Soil depth (cm)	C accretion (g·m^−2^·year^−1^)	N accretion (g·m^−2^·year^−1^)	P accretion (g·m^−2^·year^−1^)	Reference
N_2_-fixing shrubs								
Texas, USA	230	*Prosopis glandulosa*	40	0–100	17	1.2	–	Jackson et al. (2002)
Arizona, USA	370	*Prosopis velutina*	∼100	0–20	10		–	Throop and Archer (2008)
Arizona, USA	330–430	*P. velutina*	∼100	0–20	6–12	0.6–1.3	–	Wheeler et al. (2007)
Texas, USA	660	*P. glandulosa*	30	0–100	−25	−3.3	–	Jackson et al. (2002)
Texas, USA	715	*P. glandulosa*	0–130	0–15	–	0.8–1.1	–	Boutton et al. (2010)
Texas, USA	645–850	*P. glandulosa*	50–120	0–20	4–9	0.9–2.2	0.003–0.007	Geesing et al. (2000)
Texas, USA	710	*Acacia farnesiana*	5–50	0–10	239		–	Bush (2008)
Texas, USA	716	*P. glandulosa*	14–86	0–15	16		–	Creamer et al. (2011)
Texas, USA	716	*P. glandulosa*	10–130	0–15	10–30	1–3	–	Liao et al. (2006)
Texas, USA	720	*P. glandulosa*		0–20	12–22	1.9–2.7	–	Archer et al. (2001, 2004)
Texas, USA	720	*P. glandulosa*	50–77	0–10	8–23	0.9–2.0	–	Hibbard et al.(2001)
Zambia, Africa	753	*D. cinerea*	7–30	0–10	12–16	1.3–2.0	n.s.^2^	This study
Texas, USA	840	*P. glandulosa*	75–100	0–100	−61–82	−3.8 –5.7	–	Jackson et al. (2002)
Washington, USA	888	*Cytisus scoparius*	10–15	0–10	15–23	1.8–2.8	–	Haubensak and Parker (2004)
Iberian peninsula	700–1250	*Cytisus balansae*	19	0–15	42	6.6	–	Montane et al. (2007)
Nonfixing shrubs								
Texas, USA	277	*Larrea tridentate*	>50	0–100	4	−0.3	–	Jackson et al. (2002)
Texas, USA	322	*Atriplex canescens*	>50	0–100	26	−5.8	–	Jackson et al. (2002)
Utah, USA	360	*Artemisia tridentata*	0–400	0–10	30	1	–	Neff et al. (2009)
North Dakota, USA	400	Several shrub species^1^	17–42	0–15	18	1.7	–	Springsteen et al. (2010)
Texas, USA	835	*Juniperus virginiana*	35–75	0–10	3–16	0.2–1.2	–	McKinley and Blair (2008)
Iberian peninsula	700–1250	*Juniperus comunis*	32	0–15	28	2.8	–	Montane et al. (2007)
Texas, USA	1070	*Juniperus* spp.	40	0–100	−81	−6	–	Jackson et al. (2002)

1 *Amelanchier alnifolia, Shepherdia argentea, Rhamnus cathartica, Symphoricarpos occidentalis*.

1 With nonsignificant P accretion in time.

**Figure 1:**
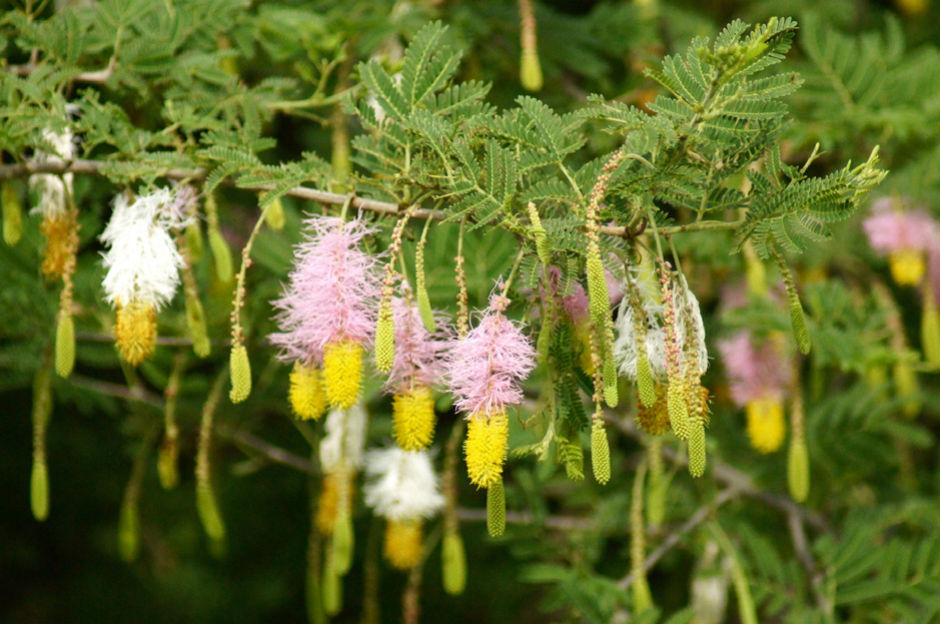
Inflorescence of the study species *Dichrostachys cinerea*.

Here, we present a study of the effects of woody encroachment on soil nutrient availabilities and carbon sequestration, as well as potential feedbacks upon encroaching shrubs. The work was conducted in a mesic savanna habitat in Zambia that was encroached to varying degrees by the leguminous thorny shrub *Dichrostachys cinerea* (Fig. 1). This species is widely distributed in subtropical and tropical Africa and can be found in Arabia, tropical Asia, America, and Australia (PROTEA 2014). It is an important woody encroacher in many African savannas (Lock 1993; Roques et al. 2001; Moleele et al. 2002; Tobler et al. 2003; Hagenah et al. 2009; Eldridge et al. 2011). In the Kafue Flats, the species only began to spread about 30 years ago (Blaser 2013), and the pre-encroachment conditions have been described in detail (Douthwaite and Van Lavieren 1977; Ellenbroek 1987).

The main aim of the study was to quantify the effects of woody encroachment on soil N, P, and C pools, and availabilities of N and P to *D. cinerea* shrubs and to the understory vegetation. Based upon known effects of leguminous shrubs in other savanna ecosystems, we developed the following working hypotheses concerning changes in soil conditions along gradients of increasing shrub age and cover:

1. N and P pools correlate positively with the age and cover of *D. cinerea* in sample plots (cf. Blaser et al. 2013);
2. Soil C pools also correlate positively with shrub age and cover;
3. Additional soil N is derived largely from atmospheric N fixation, with the consequence that *δ*^15^N correlates negatively with shrub age and cover;
4. Additional soil C is derived from *D. cinerea,* with the consequence that *δ*^13^C concentrations correlate negatively with shrub age and cover (reflecting the fact that *D. cinerea* has a C_3_ metabolism while savanna grasses are C_4_ plants);
5. Additional soil P comes from the surrounding surface soil, with the consequence that soil P pools in surrounding grasslands correlate negatively with shrub age;
6. Like some other leguminous species, *D. cinerea* shrubs have a higher root phosphatase activity than the competing plants (cf. Houlton et al. 2008; Olde Venterink 2011);
7. Because *D. cinerea* shrubs increase soil P pools, there is no negative feedback of encroachment upon N-fixing activity. As a consequence, foliar N/P ratios and N fixation rates do not change significantly along gradients of shrub age and cover.

## Materials and Methods

### Study site and selected shrub cover and age gradients

The study was conducted in the Lochinvar National Park (LNP, ∼410 km^2^) in Central Zambia (15°52′S, 27°14′E), which is located within a vast floodplain ecosystem known as the Kafue Flats. The park is a former cattle ranch in which larger carnivores were systematically exterminated in the first part of the 20th century, but wild herbivores were protected. By far the most abundant of these is the endemic Kafue lechwe, *Kobus leche kafuensis* Gray 1850 (Ellenbroek 1987). Annual precipitation is 753 mm (2000–2011 mean) and is highly seasonal, with almost all rain falling in the wet season (November–April). The mean annual temperature is 20.6°C (Ellenbroek 1987; Mumba and Thompson 2005).

The vegetation of the area can be divided into three main zones, which are strongly related to the flooding regime: seasonally inundated floodplain grasslands, the termitaria zone above the high flood line, and woodlands (Ellenbroek 1987). In the past 30 years, the termitaria and floodplain grasslands of LNP have experienced a significant increase in woodland and shrub cover – by the exotic *Mimosa pigra* in relatively wet zones of the floodplain and by the native *D. cinerea* and several native *Acacia* species in drier areas (Chabwela and Mumba 1998; Mumba and Thompson 2005; Genet 2007; Blaser 2013). These changes may be related, at least in part, to the construction of two dams in the Kafue River, which significantly altered the hydrological regime of the Kafue Flats from about 1980 onward (Blaser 2013).

In the area encroached by *D. cinerea*, we selected 20 sites (10 × 10 m) representing a cover gradient from open grassland (0% cover) to dense thicket (100% cover, corresponding to 3600 shrubs ha^−1^). We also selected 20 sites along a gradient of shrub age ranging from seven to 30 years (see below for method of determining age). Each site along the age gradient consisted of two paired plots, one located under an isolated shrub and the other a reference plot located in grassland 7 m away from the *D. cinerea* stem. Many other studies have used cover as a surrogate for age; however, we chose to study the two variables separately, because cover can be affected by other site factors such as soil nutrient availability. All sites along both gradients had similar soil and hydrological conditions. There was no obvious spatial pattern in age and cover of *D. cinerea*, and no other woody species were present.

### Determination of shrub age

**Figure 2:**
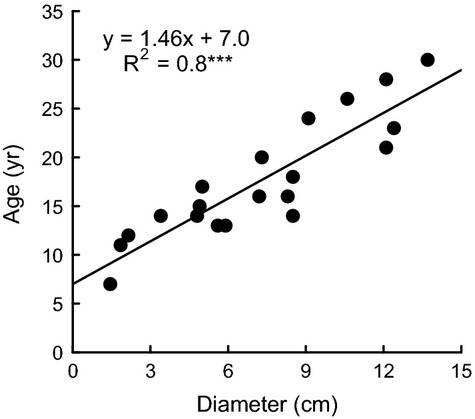
Linear relationship between *Dichrostachys cinerea* tree age, based on number of tree rings, and basal diameter, in a mesic savanna in Zambia.

At the end of the study, all shrubs along the age gradient were cut, and sample disks were taken at approximately 10 cm above ground in early April 2011. Slices were dried and polished on a belt sander with four grades of grit, to a high standard of clarity. We measured the basal diameter of the slices and examined them under a light microscope. *D. cinerea* does not have well-defined annual rings. However, for *Acacia* species growing in climatic zones with a single wet season, the number of parenchyma bands has been shown to approximate the age of the tree (Gourlay and Kanowski 1991). According to Neumann et al. (2001), the anatomy of *D. cinerea* wood is similar and can hence be included in the *Acacia* type. We therefore counted the number of continuous marginal parenchyma bands in our samples and treated this number as the shrub age. Linear regressions showed that the age of each shrub was positively related to the basal diameter (Fig. 2).

In each plot along the cover gradient, we measured the basal diameter of the thickest shrub at 10 cm above ground (assuming that this was the oldest individual) and estimated its age based on the relationship in Figure 2.

### Soil nutrient pools and availabilities

Surface soils of all sites (5.0 cm diameter cores, top 10-cm soil) were sampled during two periods in the early and mid-growing season (December 2010 and February 2011). We only sampled the topsoil layer as this layer is highly influenced by plant growth, and nutrients that are considered most limiting to plant growth are strongly cycled in this layer (Jobbagy and Jackson 2001). Along the cover gradients, we collected three soil samples at 2 m from the center of each plot. Along the age gradient, we sampled three cores 20 cm away from the *D. cinerea* stem, as well as three cores 1 m away from a central point in the paired open site. The three cores per plot or subplot were pooled, and root fragments were removed by hand. Samples were weighed to calculate bulk density, and a subsample was dried to constant weight to determine water content. The dried soil samples were then ground and sieved through a 0.5-mm sieve. Total C and N concentrations were determined using a dry combustion analyzer (CN-2000; LECO Corp., St Joseph, MN). Total P concentrations were measured colorimetrically after Kjeldahl digestion using an auto-analyzer (AutoAnalyzer 3HR; Seal Analytical, Hampshire, U.K.). To calculate soil nutrient pool (volumetric measure) for each site, we multiplied values of soil bulk density with nutrient concentrations (gravimetric measure). The *δ*^13^C and *δ*^15^N values of soil were determined for all plots using a Carlo-Erba elemental analyzer (NCS-2500, Carlo Erba) coupled in continuous flow to an isotope ratio mass spectrometer (Optima, Micro-Mass).

Assuming complete and unbiased mixing in the soil, we used the *δ*^13^C values of soils and the mean (end member) *δ*^13^C values for the foliage of *D. cinerea* (C_3_) and grass (C_4_) species present at our sites to estimate the relative proportion of soil organic matter derived from C_4_ and C_3_ photosynthetic pathway sources with an isotopic mixing model:

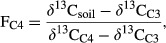

where FC_4_ is the carbon fraction derived from C_4_ sources, *δ*^13^C_soil_ is the measured *δ*^13^C of the soil sample, *δ*^13^C_C4_ is the mean *δ*^13^C of C_4_ sources, and *δ*^13^C_C3_ is the mean *δ*^13^C of C_3_ sources (Balesdent and Mariotti 1996). To estimate the *δ*^13^C value of the end members for grass and shrub foliage, foliar samples were collected along the cover gradient (see foliar nutrient contents for methods). The mean *δ*^13^C of grasses (C_4_) was −14.6 ± 0.3 ‰ (*n* = 20), whereas organic matter from *D. cinerea* (C_3_) had a *δ*^13^C value of −27.4 ± 0.1 ‰ (*n* = 19).

In December 2010 and in February 2011, we measured N and P release rates in the soil at all sites using ion-exchange resin (IER) bags. The 5 × 5 cm bags were made of a fine nylon fabric (60 *μ*mol·l^−1^ mesh width, Sefar Nitex 03- 60/35, Sefar AG, Thal, Switzerland) and contained 2 ± 0.002 g mixed-bed ion-exchange resin (Amberlite IRN 150, H^+^- and OH^−^-form, Sigma-Aldrich, Switzerland). To saturate exchange sites with K^+^ and Cl^−^ ions before use, the bags were shaken for 2 h in 2 mol·l^−1^ KCl and thoroughly rinsed with distilled water.

In the field, each bag was inserted in the soil at 5 cm depth in a 45% slant incision made with a knife and closed carefully thereafter. Four bags were set out at each site. Along the cover gradient, bags were placed 2 m from the center of each plot, along the age gradient 1 m away from the center of the plot in open sites and 20 cm away from the stem of the isolated *D. cinerea* shrubs. The bags set out in December were removed after 50 days, while those set out in February were removed after 28 days. On removal, all bags were cleaned with distilled water, dried, and stored in zip-bags until extraction. Resin bags were extracted for 1 h in 30 mL 1 mol·l^−1^ KCl solution. The extraction solution was then analyzed colorimetrically for PO_4_^3−^, NO_3_^−^, and NH_4_^+^ using a continuous flow analyzer (AutoAnalyzer 3HR; Seal Analytical). Mean daily N and P adsorption rates were calculated per site.

To estimate net N mineralization and the inorganic P pool, we collected three pairs of soil cores per plot along the *D. cinerea* cover and age gradients (5.0-cm diameter cores, top 10-cm soil) in February 2011. One core of each pair was taken for extraction and drying, while the other was incubated to measure nitrogen mineralization. In situ incubations were not possible because of high risk of flooding at some sites. The tubes containing the cores were therefore closed with plastic lids and incubated for 28 days in soil of an ex situ termitaria plot; holes in the tubes above the soil enabled gas exchange with the air. Inorganic N pool (NH_4_^+^ and NO_3_^−^) was determined by extracting a fresh equivalent of 5 g dry soil with 50 mL of 0.2 mol·l^−1^ KCl for 1 h. The inorganic P pool was determined by extracting a fresh equivalent of 5 g dry soil with 50 mL Bray-II extraction solution for 1 h (Bray and Kurtz 1945). All extractions were performed within 12 h of collection of the soil cores, and extracts were stored frozen until further analysis. Concentrations of NH_4_^+^, NO_3_^−^, and PO_4_^3−^ in the extracts were measured colorimetrically using a continuous flow injection analyzer (AutoAnalyzer 3HR; Seal Analytical). Soil pH was determined in the KCl extracts. Net N mineralization was calculated as the difference between extractable N at the start and at the end of the incubation period (Olff et al. 1994).

### Foliar nutrient contents and plant traits

Nutrient concentrations as well as *δ*^13^C and *δ*^15^N values in *D. cinerea* biomass and the above-ground grass biomass were determined along the *D. cinerea* cover gradient in the 2011–2012 growing season. To avoid possible sampling bias, we collected 10 fully expanded *D. cinerea* leaves from several heights in the canopy. We clipped the herbaceous biomass in a 50 × 50 cm square at ground level. Herbs and dead grass biomass was removed, and biomass samples were dried until constant weight. Dry samples were ground to powder, after Kjeldahl digestion analyzed for total N and P contents by means of a continuous flow injection analyzer (AutoAnalyzer 3HR; Seal Analytical). *δ*^13^C and *δ*^15^N values were determined as described above for soil samples.

We used the ^15^N natural abundance method to estimate the activity of symbiotic N_2_ fixation of *D. cinerea*. The percentage of N derived from the atmosphere (*NdfA*) was estimated following Amarger et al. (1979):

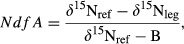

where *δ*^15^N_leg_ is the measured ^15^N abundance in the N_2_-fixing legume species, *δ*^15^N_ref_ is the mean *δ*^15^N value measured in non-N_2_-fixing reference species growing in the same location, and B is the abundance of ^15^N in a legume individual that obtains all its nitrogen from N_2_ fixation. As our sites had been chosen to contain no woody species other than *D. cinerea*, the leaf material for these analyses (leaves of *D. cinerea* as well as those of other leguminous and nonleguminous reference trees or shrubs) was collected from other sites in the same general habitat, where *D. cinerea* co-occurred with other woody species of similar size. The samples were collected in March 2011. *D. cinerea* had significant lower foliar *δ*^15^N values than the most common nonfixing reference species *Combretum imberbe* (pairwise *t*-test, *t* = *−*7.582, df = 3, *P* = 0.005) and *Diospyros senensis* (pairwise *t*-test, *t* = *−*6.915, df = 4, *P* = 0.002), which is the essential basis for determining N fixation by the natural abundance method (Boddey et al. 2001). We used the mean value of both reference species (4.85 ± 0.51‰) to calculate *NdfA* for *D. cinerea*. The parameter B was set to the lowest detected *δ*^15^N value of a legume (*−*0.0269 ‰ in *D. cinerea*), following Hansen and Vinther (2001) and Cech et al. (2010).

In March 2011, we measured the root phosphomonoesterase (PME) activity of *D. cinerea* and 10 other plant species that commonly occur with *D. cinerea* – four legumes (*Acacia nilotica*,*Cassia mimosoides, Crotalaria sp*., *Sesbania sp.),* four grasses (*Dichanthium insculptum, Digitaria milanjiana, Panicum novemnerve, Sporobolus ioclados*), and two forbs (*Epaltes alata, Hygrophila auriculata*). The root samples were collected in 10 sites containing small *D. cinerea* shrubs (∼5 years old) and located in the same general study area as the plots used in the main study. Small shrubs were chosen for practical reasons but were assumed to show similar differences in phosphatase activity than more mature individuals because phosphatase activity is tightly controlled by biologic demand of P (Allison et al. 2007; Olde Venterink 2011; Brooks et al. 2013) and we are not aware of possible effects of plant age (young vs. mature) for this enzyme activity. To collect roots, we carefully dug a hole around targeted plant and removed plants and roots together with a block of soil, which was removed by gentle shaking. Plants were then placed in water and transported to our field laboratory, where roots were washed gently to remove remaining soil. For *D. cinerea,* holes were dug *ca*. 1 m deep to obtain fine root material from different soil depths. Tap roots could not be followed to the end as they all reached deeper than 1 m. Within 12 h after sampling, three analytical replicates of cleaned root pieces (100 mg) were incubated in reaction tubes with 5 mL of a 5 mmol·l^−1^ *p*-nitrophenyl phosphate (pNPP) solution buffered at pH 6 (Tabatabai and Bremner 1969; Olde Venterink 2011). After 1 h of shaking at room temperature, the reaction was stopped by adding 0.5 mL of each test solution to 6.5 mL of 2 N NaOH. The absorbance of the solution was measured at 410 nm using a spectrophotometer (HACH, Loveland, CO) and converted into the amount of *p*-nitrophenol (pNP) released from the substrate. Phosphomonoesterase activity was then expressed as *μ*mol *p*-nitrophenol produced per g fresh root mass and hour and hence as *μ*mol pNPP cleaved.

### Calculations and statistical analysis

All plant and soil variables along the gradients were analyzed using linear regression with shrub cover and shrub age as the independent variable. For analyzing the age gradient data, we used both the absolute values around the shrubs, and also relative values represented by the difference between encroached site (20 cm around the shrub trunk) and the paired open reference site (referred to here as the “shrub effect”). In calculations using total C, N, and P pools, we used the mean values of both measurement periods (December 2010 and February 2011).

One of the IER bags yielded an exceptionally high value for resin-adsorbed P (>mean + X-STD), and this was omitted in calculating the mean site value. We also excluded this site from the extractable P regression analysis as the data were unusually high, probably due to dung or urine deposition from herbivore. Another site was completely omitted from the analyses, as a hippopotamus died and decomposed there during our study, and hence, the measured soil variable could no longer be ascribed to the effect of the tree.

Comparisons between species for PME activity were computed using one-way analysis of variance (ANOVA). Differences in N fixation of *D. cinerea* compared with nonfixing reference plants were analyzed using paired *t*-tests. When necessary, data were log-transformed to fulfill assumptions of normality and homogeneity of variance. All statistical analyses were conducted using R version 2.10.1 (R Core Team 2012).

## Results

### Soil nutrient pools and availabilities

**Figure 3:**
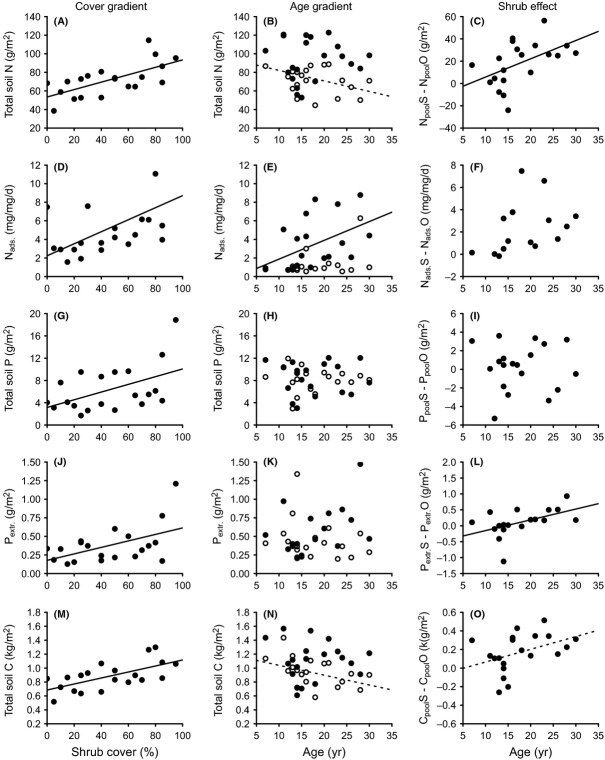
Soil N, P, and C pools, N adsorption to resin, and soil extractable P along gradients of *Dichrostachys cinerea* shrub cover or age in a mesic savanna in Zambia. Data points in the first two columns of graphs (cover gradient and age gradient) represent absolute amounts. In the age gradient: solid symbols are sites under the shrub canopy, open symbols are the paired reference sites outside the canopy. Data points in the third column (shrub effect) represent the difference in soil variables of encroached sites from the age gradient compared with adjacent reference sites. Soil samples were taken from the top 10-cm soil in December 2010, except for soil extractable P in February 2011. Solid lines represent significant linear regressions (*P *<* *0.05), and dotted lines represent a marginal significant trend (0.05 < *P *<* *0.1). *R*^2^ values and significance levels are displayed in Table 2.

The total soil N pool increased significantly along the cover gradient, with the regression line rising from 47.8 g·m^−2^ in the absence of *D. cinerea* to 98.2 g·m^−2^ at a cover of 100% (Fig. 3A; Table 2). In contrast, the N pool was not significantly related to shrub age (Fig. 3B; Table 2). However, the “shrub effect” (i.e., the difference in soil N pool beneath a tree and in adjacent grassland; Fig. 3C) did increase along the age gradient, yielding an average accretion rate of 1.6 (1.3–2.0) g·N·m^−2^·year^−1^. In the nonencroached reference sites along the age gradient, total N pools tended to decrease along the age gradient, through the result was only marginally significant (Fig. 3B). Resin-adsorbed N increased significantly with shrub cover and age, but there was no significant age effect when comparing encroached with adjacent grassland sites (Figs. 2D, 3D–F). Net N mineralization showed a quadratic relationship with shrub cover and age, with rates being lowest at intermediate values (Table 2). We found no significant patterns for extractable N and *δ*^15^N values along either gradient (Table 2).

**Table 2:** Results of linear regressions (*R*^2^ values and significance levels) for several foliar and soil variables in top 10-cm soils against gradients of *Dichrostachys cinerea* shrub cover or age. The “shrub effect” represents the difference in soil variables of encroached sites compared with adjacent reference plots. If two values are shown, the first is for the December 2010, and the second for the February 2011 measurement period. For total C, N, and P as well as total nutrient ratios, mean values of the December 2010 and February 2011 measurements were used. Sample size was *n* = 20 for all variables apart for adsorbed N and P along the age gradient *n* = 16–20 and for total P and extractable P along the age gradient *n* = 19.

Soil variables	Cover gradient	Age gradient				
		Absolute values				Shrub effect
		Encroached	Reference plots			
Total N (g·m^−2^)	0.44**	n.s.	0.16†	0.26*
Extractable N (g·m^−2^)	n.s.	n.s.	n.s.	n.s.
Adsorbed N (mg·g^−1^·day^−1^)	0.30*/0.33**	0.27*	n.s.	n.s.
Net. N min. (g·m^−2^·day^−1^)	0.30*, 1	0.46†, 1	0.18†	0.46*, 1
*δ*^15^N (‰)	n.s.	n.s.	n.s.	n.s.
Total P (g·m^−2^)	0.24*	n.s.	n.s.	n.s.
Extractable P (g·m^−2^)	0.26*	0.21†	n.s.	0.25*
Adsorbed P (mg·g^−1^·day^−1^)	n.s./n.s.	0.17†	n.s.	n.s.
Total C (kg·m^−2^)	0.40**	n.s.	0.18†	0.17†
*δ*^13^C (‰)	0.66***	0.56***	n.s.	0.72***
Total N:P	n.s.	n.s.	n.s.	0.15†
Extractable N:P	n.s.	n.s.	n.s.	n.s.
Adsorbed N:P	n.s./n.s.	n.s.	n.s.	n.s.
Total C:N	n.s.	0.15†	n.s.	n.s.
Total C:P	n.s.	n.s.	n.s.	n.s.
pH	n.s./n.s.	n.s./n.s.	n.s./0.20*	n.s./0.29*
Moisture (%)	n.s./n.s.	n.s./n.s.	n.s./n.s.	n.s./n.s.
Bulk density (g·cm^−3^)	n.s.	0.21*	n.s.	0.20†
Foliar variables				
*D. cinerea* N (mg·g^−1^)	0.50**, 1	–	–	–
*D. cinerea* P (mg·g^−1^)	0.56**, 1	–	–	–
*D. cinerea* N:P	0.53**, 1	–	–	–
*D. cinerea δ*^15^N (‰)	0.40**	–	–	–
*D. cinerea Ndfa* (%)	0.40**	–	–	–
Alive grass N (mg·g^−1^)	0.24*	–	–	–
Alive grass P (mg·g^−1^)	0.41**	–	–	–
Alive grass N:P	0.34**	–	–	–
Alive grass *δ*^15^N (‰)	0.38*, 1	–	–	–

n.s., nonsignificant results.

† *P* < 0.1,

* *P* < 0.05,

** *P* < 0.01,

*** *P* < 0.001.

1 Quadratic regression.

Total P and extractable P pools increased along the shrub cover gradient (Fig. 3G and J). There was also a significant positive effect of age upon extractable P beneath shrubs relative to that in reference plots (i.e., shrub effect; Fig. 3L). P adsorption to resin marginally increased along the age gradient, but not along the shrub cover gradient (data not shown, Table 2).

**Figure 4:**
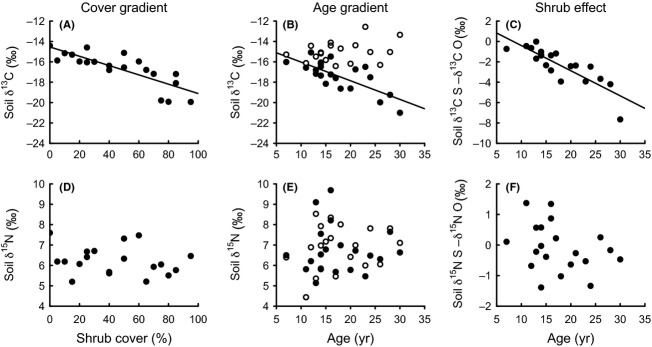
Soil *δ*^13^C and *δ*^15^N values along gradients of *Dichrostachys cinerea* shrub cover or age in a mesic savanna in Zambia. Data points in the first two columns of graphs (cover gradient and age gradient) represent absolute amounts. In the age gradient, solid symbols are sites under the shrub canopy, and open symbols are the paired reference sites outside the canopy. Data points in the third column (shrub effect) represent the difference in soil variables of encroached sites from the age gradient compared with adjacent reference sites. Soil samples were taken from the top 10-cm soil in December 2010. Solid lines represent significant linear regressions (*P *<* *0.05). *R*^2^ values and significance levels are displayed in Table 2.

The total soil C pool increased significantly with cover but not age (Fig. 3M and N). However, the shrub effect data (i.e., the difference between encroached with reference sites) did show a marginally significant increase with age, which was equivalent to an accretion rate of 14 (12–16) g·C·m^−2^·year^−1^ (Fig. 3O). In the nonencroached reference sites along the age gradient, total C pools tended to decrease with age (Fig. 3N). *δ*^13^C values decreased with both cover and age (Fig. 4A–C, Table 2) and showed that the proportion of C derived from C_3_ plant increased with shrub age in the encroached but not in the adjacent reference sites (Fig. 4B). Based upon the isotopic mixing model, we calculated that the proportion of soil C derived from C_3_ plants was <42% under dense *D. cinerea* and <50% under 30-year-old shrubs.

The N/P ratio for soil pools increased with shrub age, and the equivalent C/N ratio decreased with shrub cover, both trends being marginally significant (data not shown, Table 2). None of the other ratios (N/P, C/P and C/N) showed any significant patterns along either gradient (data not shown, Table 2).

Soil bulk density did not vary significantly with shrub cover, but decreased with age, leading to significantly larger differences between encroached and reference plots with increasing age (data not shown, Table 2). Plots did not differ in soil moisture. Soil pH did not vary with cover and age for plots around trees. However, pH in adjacent grassland plots increased in one measurement period, leading to larger (negative) differences between encroached and reference plots with increasing age (Table 2). We found no relationships between pH and total and extractable P pools (data not shown).

Overall, our results confirmed that in this study system a cover gradient accurately reflects dynamics along an age gradient. Linear regression showed that higher cover was positively related to age (*R*^2^ = 0.42, *P* = 0.002).

### Foliar nutrients and δ^15^N values of *D. cinerea* and understory grass biomass

**Figure 5:**
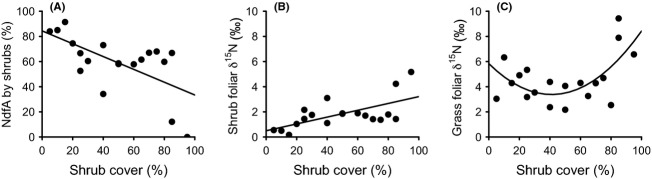
Foliar N and P concentrations, and N/P ratios, of the shrub *Dichrostachys cinerea* and of understory alive grass biomass, along a *D. cinerea* cover gradient in a mesic savanna in Zambia, in January 2012. Quadratic and linear regressions are significant (*P *<* *0.05). *R*^2^ values and significance levels are displayed in Table 2.

N and P concentrations and N/P ratios of *D. cinerea* foliage showed quadratic relationships with shrub cover, with N and P values being lowest and N/P ratios peaking at intermediate shrub cover (Fig. 5A–C, Table 2). Foliar N and P concentrations in understory grass biomass (living material) increased significantly along the shrub cover gradient, while the N/P ratio decreased (Fig. 5D–F, Table 2).

**Figure 6:**
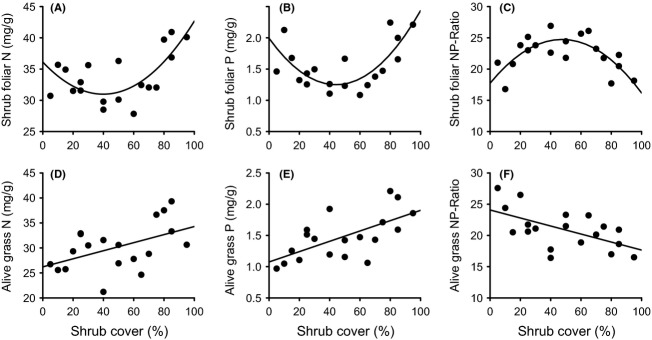
*Dichrostachys cinerea* and grass foliar *δ*^15^N and % N derived from atmosphere (*NdfA*) along a cover gradient of *D. cinerea* shrubs. Quadratic and linear regressions are significant (*P *< 0.05). *R*^2^ values and significance levels are displayed in Table 2.

Foliar δ^15^N values increased and *NdfA* of *D. cinerea* shrubs decreased along the cover gradient (Fig. 6A and B). Changes in *NdfA* were unrelated to changes in soil pH (data not shown). Foliar δ^15^N values of the understory grasses followed a quadratic relationship with lowest values occurring at intermediate shrub cover (Fig. 6C).

### Root phosphomonoesterase (PME) activity

**Figure 7:**
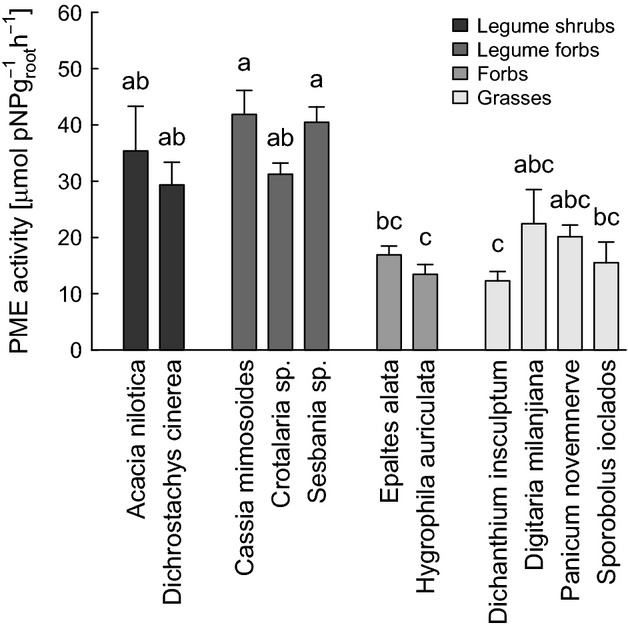
Root phosphomonoesterase (PME) activity of 11 plant species from a mesic savanna in Zambia. Error bars show standard errors of 4–12 samples. Species not connected by same letter are significantly different (Tukey HSD test).

As a group, the legumes had significantly higher PME activity than either grasses or forbs (ANOVA, *P* < 0.001, *F* = 36.4). However, in comparisons among individual species, PME activity of *D. cinerea* did not differ significantly from the values for any other species except *Hygrophila auriculata* and *Dicanthium insculptum*, which were lower (Fig. 7).

## Discussion

### Increases in soil N and P pools under the encroaching shrubs

Encroachment of the N-fixing shrub *D. cinerea* in our Zambian mesic savanna increased topsoil pools of both N and P. This finding is consistent with results from other African savanna ecosystems, where sites encroached by individual trees have been compared with open grassland (Ludwig et al. 2004; Hagos and Smit 2005). The difference in soil N pools between paired sites with and without *D. cinerea* increased with shrub age, indicating that the net nutrient accumulation was induced by the encroaching shrub species (Fig. 3). Furthermore, the rates of nutrient accretion (1.3–2.0 g·N·m^−2^·year^−1^) correspond well with those reported elsewhere, although these are only available for a few species, mainly from sites in the USA (Table 1). The finding that P accumulates in the topsoil along our gradients is consistent with similar studies of N-fixing woody species in other African savannas (Ludwig et al. 2004; Sitters et al. 2013). Thus, our study contributes to a growing body of evidence that N-fixing woody plants might obtain P from sources not available to grasses. Despite having a higher P requirement than grasses (Binkley et al. 2000; Vitousek et al. 2002), therefore, they may not suffer from P limitation induced by their own growth (i.e., there is no negative feedback).

In a recent meta-analysis, Blaser et al. (2013) showed that N-fixing woody plants increase soil N more than other woody plants. In this study, the increase in soil N under *D. cinerea* is probably due to shrubs producing more biomass than competing grasses and to their capacity to fix N. Although we did not measure litter production, foliage production by *D. cinerea* was almost three times higher than that of grasses (658 ± 117 g·m^−2^ mean of six 14- to 20-year-old *D. cinerea* trees vs. 237.6 ± 50 g·m^−2^ for similar-sized grassland plots, Blaser 2013). Furthermore, the fact that roots of seedlings and young shrubs contained nodules, while foliar *δ*^15^N values were significantly lower than for nonfixing shrubs, are clear indications that the trees were fixing atmospheric N_2_. On the other hand, soil *δ*^15^N values did not decrease along the gradient as would be expected if the source of the increase in soil N pools was N fixation (Fig. 4, Boutton and Liao 2010). However, this effect might have been neutralized by other processes affecting the ^15^N concentration, such as N pumping from deeper soil layers and isotope fractionation during litter decomposition (Hobbie and Ouimette 2009; Boutton and Liao 2010) and denitrification (Robinson 2001; Garten et al. 2008). As our sites were inundated for several weeks each year, denitrification may have been a significant process, with higher rates under denser stands of *D. cinerea* where N concentrations and probably nitrate were higher (Fig. 3D and M; Davidson and Swank 1986; Olde Venterink et al. 2002; Seitzinger 1994).

Contrary to our initial hypothesis, the increases in both total P and extractable P in the topsoil of encroached areas were probably not due to lateral transport in widely spreading roots, as we found no evidence of depletion of either total or extractable P in the nonencroached reference sites (Fig. 3H and K). This conclusion is consistent with results obtained along a gradient of increasing *Acacia zanzibarica* density in moist savanna in Tanzania, where trees were also not found to deplete extractable P pools in topsoil beyond the canopy (Sitters et al. 2013). An alternative explanation could be a higher root phosphatase activity of this leguminous species compared with other plants (Houlton et al. 2008; Olde Venterink 2011). However, despite generally higher levels of PME activity in leguminous species, the differences between *D. cinerea* and other species were mainly not significant (Fig. 7). It is also unlikely that N fixation increased P availability by acidifying the rhizosphere, as we found no increase in pH along the gradients and no relationship between pH and soil P concentration. This leads us to conclude that the increases in both total P and extractable P were mainly due to *D. cinerea* having deeper roots than the competing grasses (pers. observation, Us Forest Service 2013), enabling shrubs to take up P from lower soil layers. Any additional P acquired in this way is initially incorporated into plant tissues, but becomes available in the topsoil as litter falls to the ground and decomposes (Marsh et al. 2000; Jobbagy and Jackson 2001; Jackson et al. 2002; McCulley et al. 2004). To test this hypothesis, future studies should sample soils in deeper layers and obtain more detailed measurements on rooting depth of *D. cinerea*.

In the nonencroached reference sites, total N and C pools tended to decrease along the age gradient (Fig. 3B and N). One reason for these unexpected effects, albeit only marginally significant, could be that shrubs were in some way altering soil conditions at a distance. However, this seems unlikely, given that we observed no lateral accumulation of P (see above), while N and C contents in soil of open sites were highly correlated which indicates that the N originated from organic matter. A second possibility is that the temporal spread of shrubs was confounded with a spatial pattern in soil conditions, with more recently colonized sites containing more organic matter. This pattern could have reflected small differences in topography, with the soil in moister depressions having a higher organic content (Gregorich et al. 1998), although we have no data to test this possibility. Such a confounding pattern could explain why we found no absolute increase in soil nutrients with age, as has been reported in other studies (e.g., Geesing et al. 2000; Ludwig et al. 2004; Throop and Archer 2008). Not finding enhanced total C pools in encroached plots along the age gradient could be related to the fact that our shrubs were still relatively young (cf. other studies in Table 1) and effects along the gradient might have been too small to overcome initial differences in soil C pools among sites. This would also explain why the observed replacement of grass-derived C with shrub-derived C with increasing encroachment age (Fig. 4B) was not translated in a detectable enhanced total C pool (Fig. 3N). However, because we used a paired plot approach, we are confident that possible heterogeneities in site conditions do not affect our main conclusions.

### Plant soil feedback

Two important results emerge from the foliar analyses of shrubs and grasses. First, the analyses of *D. cinerea* show highly significant unimodal patterns in N and P concentrations, which are lowest at intermediate shrub cover, while the N/P ratio peaks at intermediate cover (Fig. 5A–C). These patterns suggest a negative feedback at intermediate shrub cover and a positive feedback when shrubs reach a certain density or age (because denser plots often contained older trees, age and cover are positively correlated; *R*^2^ = 0.42 *P *=* *0.002). Second, N and P concentrations in grass samples increase along the cover gradient (Fig. 5D and E), suggesting that shrubs in some way improve nutrient availabilities for understory plants. To explain these patterns, we suggest that a shrub colonizing a grass-dominated site has a good P supply at first, perhaps because it rapidly establishes a deep root system or can utilize organic forms of P better than competing grasses (or both, Sitters et al. 2013). With increasing shrub age and density, the easily accessible P fractions gradually become depleted, which negatively affects P uptake and consequently also N fixation (Almeida et al. 2000). As a deep root system continues to develop, however, and as litter is returned to the soil surface, the supply of organic P and N in the topsoil increases (Sitters et al. 2013), while competition from understory grasses is reduced through shading (Blaser 2013). Through these processes, the P supply for shrubs improves once the shrub cover has reached about 50%. Meanwhile, N availability also increases again. However, as foliar *δ*^15^N values indicate reduced N fixation rates in plots with a dense shrub cover, we suppose this effect is mostly due to increased production and accumulation of organic matter (Fig. 6A and B). Thus, of the two main factors known to control N fixation (Vitousek et al. 2002; Pons et al. 2007), increased soil N availability is likely to have been more important than reduced P availability (cf. Fig. 3). Plants in the understory also benefit from the increased nutrient supply under high shrub cover, although their growth becomes increasingly limited by factors such as shortage of water or light (Blaser 2013). Moreover, the decreasing N/P ratio in the grasses with increasing cover indicates that N becomes relatively more limiting than P in the denser sites (Cech et al. 2008).

Overall, we found no evidence that *D. cinerea* negatively affects its own nutrient supply, and also no indication that encroachment is part of a cyclical process that will eventually return the ecosystem to grassland. In the altered hydrological conditions of the Kafue Flats, however, *D. cinerea* might gradually be replaced by nonfixing trees, converting the former grasslands into woodland communities, as has occurred in other savanna ecosystems (Archer et al. 1988).

### Effects on carbon sequestration

The question of how the carbon balance of an ecosystem is affected by shrub encroachment has proved difficult to answer, and the evidence remains controversial. Jackson et al. (2002) found that woody encroachment in grasslands increased soil C and N stocks in drier regions, but decreased them in regions with mean annual precipitation (MAP) greater than *c*. 500 mm·year^−1^ (Table 1). However, Barger et al. (2011) tested the robustness of the pattern with MAP and concluded that while changes in soil C pools with tree encroachment were inversely related to MAP, responses to shrub encroachment were highly variable and unrelated to MAP. This conclusion was supported by a recent meta-analysis (Eldridge et al. 2011), which confirmed that effects of encroachment (by both trees and shrubs) on soil C pools were not dependent upon rainfall. Moreover, we found 15 studies with data from 21 different locations that could be used to calculate soil C accretion rates (Table 1). In this sample, the rates ranged from losses of −80 g·C·m^−2^·year^−1^ to accumulations of 239 g·C·m^−2^·year^−1^ (mean 21 g·C·m^−2^·year^−1^) and were unrelated to MAP (Table 1). The results presented in our study also indicate that encroachment in mesic savanna ecosystems can be associated with positive soil C and N balances. Furthermore, we only studied the top 10-cm soil, and C sequestration likely takes place in deeper soil layers as well (Jackson et al. 2002), perhaps serving as a C sink for centuries (Jobbagy and Jackson 2000).

A second, related problem concerns how the capacity of ecosystems to sequester C may be affected by rising concentrations of atmospheric CO_2_. Based upon a meta-analysis, Van Groenigen et al. (2006) concluded that C storage in the soil could only increase if N inputs were to rise substantially above present levels (<30 kg·N·ha^−1^·year^−1^ in the US and Europe). However, in encroached soils, we recorded an increase of 1.3–2.0 g·C·m^−2^·year^−1^ with no external addition of N, suggesting that N-fixing species such as *D. cinerea* have the potential to overcome this particular constraint upon carbon accumulation (Hungate et al. 2003). Furthermore, P limitation is also unlikely to become a constraint, in our study system at least, because of the species' ability to mobilize P, which we assume originates from deeper soil layers. Thus, as shrub encroachment in mesic savannas has increased and is predicted to further increase by 70% with CO_2_ concentrations doubling (Bond et al. 2003), this ecoregion might continue to act as a carbon sink for many decades.

## Conclusions

Both N and P pools in the soil increased along gradients of shrub age and cover, suggesting that N fixation by *D. cinerea* did not reduce the P supply. This in turn suggests that continued growth and carbon sequestration in this mesic savanna ecosystems are unlikely to be constrained by nutrient limitation. Further studies would be needed to determine whether this finding applies for other woody species and in other areas. However, if our results for *D. cinerea* are representative for other encroaching N-fixing trees in savannas, these ecosystems could become substantial C sinks for several decades.
